# Decellularized rat submandibular gland as an alternative scaffold for dental pulp regeneration

**DOI:** 10.3389/fbioe.2023.1148532

**Published:** 2023-04-21

**Authors:** Yuanyuan Shi, Yingxin Wang, Zhaochen Shan, Zhenhua Gao

**Affiliations:** Outpatient Department of Oral and Maxillofacial Surgery, Capital Medical University School of Stomatology, Beijing, China

**Keywords:** decellularization, extracellular matrix, dental pulp regeneration, tissue engineering, submandibular gland, scaffold

## Abstract

**Introduction:** Decellularized extracellular matrix has been recognized as an optimal scaffold for dental pulp regeneration. However, the limited amount of native dental pulp tissue restricts its clinical applications. The submandibular gland shares some basic extracellular matrix components and characteristics with dental pulp. However, whether decellularized submandibular gland extracellular matrix (DSMG) can be used as an alternative scaffold for dental pulp regenerative medicine is unclear.

**Methods:** Thus, we successfully decellularized the whole rat submandibular gland and human dental pulp, and then conducted *in vitro* and *in vivo* studies to compare the properties of these two scaffolds for dental pulp regeneration.

**Results:** Our results showed that extracellular matrix of the submandibular gland had great similarities in structure and composition with that of dental pulp. Furthermore, it was confirmed that the DSMG could support adhesion and proliferation of dental pulp stem cells *in vitro*. *In vivo* findings revealed that implanted cell-seeded DSMG formed a vascularized dental pulp-like tissue and expressed markers involved in dentinogenesis and angiogenesis.

**Discussion:** In summary, we introduced a novel accessible biological scaffold and validated its effectiveness as an extracellular matrix-based tissue engineering scaffold for dental pulp regenerative therapy.

## 1 Introduction

Dental pulp is a highly vascularized and innervated connective tissue containing a population of stem cells that reside in the root canal surrounded by hard tissue. The physiological properties and homeostasis of dental pulp are closely connected to the extracellular matrix (ECM) microenvironment niche (Linde, 1985). Dental pulp is vulnerable to bacterial invasion or trauma, resulting in irreversible pulpitis or pulp necrosis. The current conventional endodontic treatment, root canal therapy, compromises the immune defense function and changes the physical properties of dentin, making it susceptible to infection or fractures, resulting in eventual tooth loss (Siqueira, 2001). Dental pulp regeneration has been proposed to replace mechanical barriers with biological tissue in order to improve clinical outcomes, emerging as a promising alternative strategy for future clinical applications (Law, 2013; Duncan and Cooper, 2019).

Currently, the dental pulp regeneration procedure has reached the clinical trial phase for therapeutic applications in humans. The outcomes, however, remained unpredictable (Rosa et al., 2013; Brizuela et al., 2020; Huang et al., 2020). Among these regenerative technologies, cell-based tissue engineering techniques have been used to effectively reestablish intricate pulp structure and restore tooth vitality (Sui et al., 2019). Dental pulp stem cells (DPSCs), an optimal population of cells in regenerative dentistry, have been demonstrated to be capable of producing a pulp-dentin complex and promoting angiogenesis (Gronthos et al., 2002; Huang et al., 2009). A great diversity of complicated scaffolds has been fabricated. Nevertheless, none of them could systematically regulate coordinated regeneration of the multiple tissue types involved in the functional dental pulp-dentin complex (Raddall et al., 2019). Moreover, blood vessels are difficult to induce from the small apical foramen (Lin et al., 2014; Cao et al., 2015; Rombouts et al., 2017). Thus, the lack of an available scaffold to reproduce the complex dental pulp ECM that supports cell-matrix interactions and enhances revascularization is a challenge faced by regenerative endodontics. Decellularized tissue scaffolds have long been used in regenerative medicine to provide a non-immune environment with retention of the native structure and molecular composition, as well as to preserve mechanical and biochemical properties, rendering ECM-derived scaffolds ideal candidates for pulp regenerative therapy (Taipale and Keski-Oja, 1997). Despite the current success in optimizing decellularization protocols of human, swine, or bovine dental pulp tissue, allogeneic or xenogeneic dental pulp tissue has limited availability (Song et al., 2017; Zhang et al., 2017; Alqahtani et al., 2018; Matoug-Elwerfelli et al., 2018; Alghutaimel et al., 2021; Bakhtiar et al., 2021).

The submandibular gland is a vital maxillofacial organ with an abundant ECM and basic matrix proteins similar to dental pulp (Bartel-Friedrich et al., 1999; Gao et al., 2014). Furthermore, it can be obtained in large quantities from small animals; hence, the present study aimed to evaluate decellularized ECM of the submandibular gland as an alternative scaffold for extensive pulp regeneration.

## 2 Materials and methods

### 2.1 Experimental design

**FIGURE 1:**
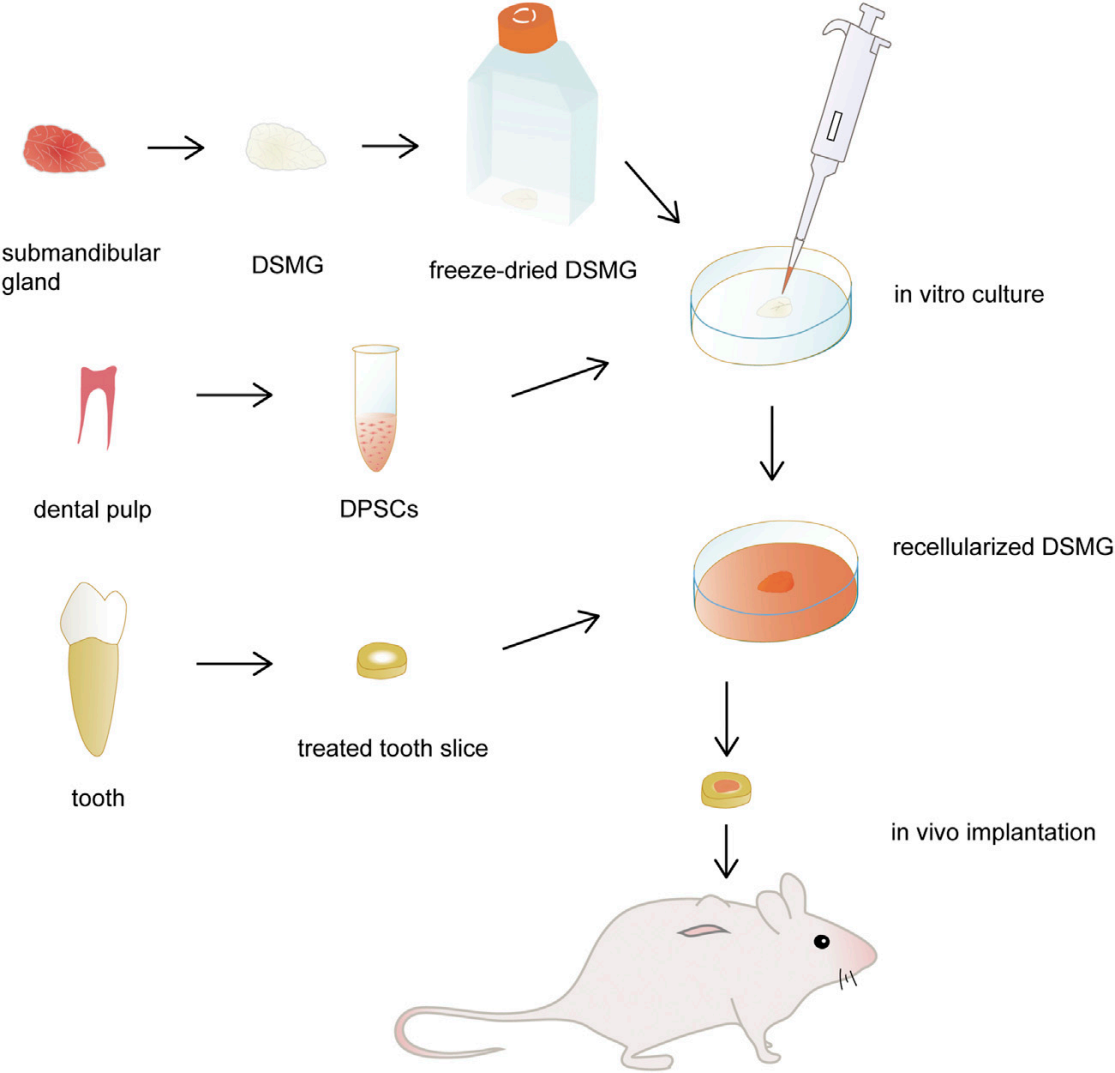
Schematic illustration of the experimental design. The submandibular glands were decellularized and then freeze-dried as DMSG scaffolds. DPSCs were seeded to the scaffolds and cultured *in vitro* for 3 days. Recellularized constructs were filled into treated tooth slices and then implanted subcutaneously into immunodeficient mice for dental pulp regeneration, with acellular constructs as controls. DSMG, decellularized submandibular gland; DPSCs, dental pulp stem cells.

The illustration of the experimental design is shown in Figure 1. Microstructure and composition of decellularized submandibular gland extracellular matrix (DSMG) were characterized. Thereafter, scaffolds seeded with DPSCs were cultured *in vitro* for 7–14 days to evaluate cell adhesion and differentiation. In addition, recellularized constructs, together with acellular scaffolds, were filled into treated tooth slices and then implanted subcutaneously into immunodeficient mice for 12 weeks to facilitate dental pulp regeneration.

### 2.2 Tissue preparation and decellularization

All animal experiments were approved by the Ethics Committee for Animal Experimentation of the Beijing Stomatological Hospital, Capital Medical University (Beijing, China), and protocols conformed to the guidelines of Animal Research: Reporting of *in vivo* Experiments. Male 8-week-old Sprague-Dawley rats were euthanized and decellularization protocols of their submandibular glands were performed as previously described (Gao et al., 2014). Briefly, samples were placed in 10% (w/v) sodium dodecyl sulfate (SDS; Sigma-Aldrich, United States) in deionized water for 32 h, with fresh SDS solution changed every 8 h. The samples were washed thoroughly with enough sterile, deionized water and then treated with 1% (w/v) Triton X-100 (Sigma-Aldrich) in deionized water for 2 h, followed by washing with PBS to remove residual detergents. Extracted teeth were obtained upon approval from the Ethics Committee of the Beijing Stomatological Hospital, Capital Medical University (permit number: CMUSH-IRB-KJ-PJ-2021-25), with informed consent of the patients. Human dental pulp was obtained from healthy third molars of individuals aged 18–25 years and then decellularized through cycles of 1% SDS solution and 1% Triton X-100 solution based on published reports (Song et al., 2017; Matoug-Elwerfelli et al., 2018). The decellularized scaffolds were sterilized via immersion in 10 mg/mL streptomycin, 10,000 U/mL penicillin G, and 25 μg/mL amphotericin B (Solarbio, China) for 12 h. The prepared scaffolds were stored at 4°C until freeze-drying.

### 2.3 Histology and immunofluorescence characterization of ECM

The obtained native submandibular glands and dental pulp tissue were fixed in 4% paraformaldehyde overnight, dehydrated with graded ethanol, embedded in paraffin, and then sectioned at 5 μm. Hematoxylin and eosin (H&E) staining was performed to characterize the ECM histology and immunofluorescence staining was conducted to label some vital ECM proteins of both tissues, which included collagen type I alpha (COL1, mouse monoclonal to collagen I; Santa Cruz, United States), collagen type III alpha 1 (COL3, mouse monoclonal to collagen 3; Santa Cruz), and fibronectin (mouse monoclonal to fibronectin; Santa Cruz). For immunofluorescence, deparaffinized sections were blocked against non-specific binding using goat serum and then incubated with diluted primary antibodies (anti-COL1, 1:200; anti-COL3, 1:200; anti-fibronectin, 1:200) at 4°C overnight. Alexa Fluor 488 goat anti-mouse IgG (ABclonal, United States) was used as a secondary antibody to visualize components specific to the ECM and DAPI was used to label nuclei. The stained sections were observed under a fluorescence microscope (Olympus BX61, cellSens Standard software).

### 2.4 Masson’s trichrome and sirius red staining of ECM

Tissue sections of the DSMG and decellularized dental pulp (DDP) were stained using a Masson’s trichrome staining kit (Solarbio) containing Biebrich scarlet-acid fuchsin and aniline blue solution according to the manufacturer’s recommended protocol. For Sirius red staining, sections were deparaffinized, loaded with dye solution, and incubated at 25°C for 30 min using a Sirius red/Fast green collagen staining kit (Chondrex, United States). Some of these samples were observed under a microscope (Olympus). To quantify total collagen content, other samples were then loaded with dye extraction buffer and mixed by pipetting until the color was eluted from the tissue section. In the final step, we collected the eluted dye solution and measured the absorbance at 540 and 605 nm with a spectrophotometer (SpectraMax Paradigm, Multi-Mode Microplate Reader).

### 2.5 Scanning electron microscope analysis of ECM

To compare the ultrastructure of DSMG and DDP, decellularized samples were rinsed in PBS and then fixed in 2.5% glutaraldehyde at 4°C for 24 h. The samples were then post-fixed in 1% osmium tetroxide at 25°C for 1 h, dehydrated sequentially in ethanol, dried in a critical point dryer (Quorum K850), sputter-coated with gold, and then visualized using a scanning electron microscope (SEM; Hitachi SU8100).

### 2.6 Freeze-drying of DSMG scaffolds

Prior to freeze-drying, sterilized scaffolds were precultured in alpha minimum essential medium (α-MEM; Cytiva, United States) at 37°C overnight. The scaffolds were washed with PBS and then collected in sterilized 25 cm^2^ cell culture flasks with vent caps (Corning, United States) that were pre-frozen in a freezer at −80°C for 2 h and then placed on the cold shelf of a freeze dryer (Christ ALPHA1-2LDplus). The drying protocols were performed for 24 h at an ice condenser temperature of −51°C and a vacuum of 0.07 mbar. The freeze-dried products were maintained in sealed cell culture flasks and stored at 4°C until further use.

### 2.7 Isolation and culture of DPSCs

DPSCs were isolated and cultured as described in previous studies (Gronthos et al., 2000; Karamzadeh et al., 2012). Briefly, dental pulp tissue separated from human adult third molars was cut into small pieces and then digested in an enzyme solution containing 3 mg/mL collagenase type I (Sigma-Aldrich) and 4 mg/mL dispase II (Sigma-Aldrich) for 1 h at 37°C. Single-cell suspensions were obtained by passing the cells through a 70 μm cell strainer (Corning) and then centrifuged at 1,100 rpm for 6 min. The supernatant was carefully removed with a pipette and the cell pellet was resuspended in 2 mL of α-MEM supplemented with 20% fetal bovine serum (Gibco, United States), 100 U/mL penicillin, 100 μg/mL streptomycin, 0.25 μg/mL amphotericin B, and 2 mM l-glutamine, and then incubated in a humidified incubator at 37°C with 5% CO_2_. Fresh culture medium was changed every 3 days until the adherent cells reached 80% confluence. Cells were digested using trypsin (Gibco) and passaged. Cells obtained after 3–5 passages were used in subsequent experiments.

### 2.8 Cell adhesion and proliferation in scaffolds *in vitro*

To investigate the biological activity of DSMG, DPSCs were pipetted at a density of 1 × 10^7^ cells/mL directly into lyophilized DSMG (100 μL in each scaffold) in 24-well culture plates. After incubation for 2 h, an additional 1 mL of basal medium (α-MEM containing 10% fetal bovine serum, 100 U/mL penicillin, 0.1 mg/mL streptomycin, 0.25 μg/mL amphotericin B, and 2 mM l-glutamine) was applied to each well with fresh medium changed every 2 days. Acellular lyophilized scaffolds immersed in basal medium were used as a control. The constructs were cultured at 37°C in a 5% CO_2_ atmosphere for 1, 3, 5, or 7 days. The recellularized scaffolds harvested at days 3, 5, and 7 were processed individually for SEM observation of the adhesion and morphology of DPSCs on DSMG. For further characterization of cell viability and proliferation capacity, cell counting kit solution (Cell Counting kit-8; Dojindo, Japan) was added to each well and incubated for 2 h. The supernatant was pipetted into 96-well culture plates and absorbance was measured at 450 nm with a spectrophotometer.

### 2.9 Cell differentiation in scaffolds *in vitro*

Real-time quantitative polymerase chain reaction (PCR) was performed to analyze the influence of DSMG scaffolds on the expression of odontogenic-related markers in DPSCs. DPSCs were seeded at a density of 1 × 10^7^ cells/mL into lyophilized DSMG (100 μL in each scaffold) in 24-well culture plates. The constructs were cultured at 37°C with 5% CO_2_ for 7 and 14 days *in vitro*. Total RNA was extracted from recellularized scaffolds using TRIzol Reagent (Invitrogen, United States). The cDNA was synthesized and amplified using a real-time PCR detection system (Bio-Rad CFX manager, United States). Sequences of the specific primers used are listed in Supplementary Table S1, with *GAPDH* as the housekeeping gene. The relative mRNA expression was calculated using the 2^−ΔΔCT^ method. Each measurement was performed in triplicate.

### 2.10 *In vivo* implantation and dental pulp regeneration

DSMG scaffolds were initially seeded with DPSCs and cultured *in vitro* for 3 days, bringing the cells to a rapid proliferation phase and allowing the matrix to achieve sufficient cell repopulation, as demonstrated in the *in vitro* studies. For dental pulp-dentin complex formation, treated tooth slices were required as appropriate hard tissue scaffolds supporting the above soft tissue. Tooth roots of premolars were cut into segments of 4 mm in length, and the root canals were enlarged with files. Tooth slices were treated in sequence with 17%, 10%, and 5% ethylenediamine tetra-acetic acid solution with a pH of 7.2–7.4 for 10 min, and then washed in deionized water for 10 min in an ultrasonic cleaner to remove the smear layer (Orlowski et al., 2020). For sterilization, they were soaked in sterile PBS containing 1,000 U/mL penicillin and 1 mg/mL streptomycin for 72 h, and then maintained in α-MEM at 4°C until implantation. Recellularized and acellular constructs, with six samples in each group, were inserted into the treated tooth slices and then implanted into the subcutaneous space on the dorsum of immunodeficient mice.

### 2.11 Staining and quantification

After 12 weeks, all samples were retrieved, fixed in 4% formaldehyde, decalcified with 10% ethylenediamine tetra-acetic acid solution, and then processed for H&E and Sirius red staining. For immunohistochemical analysis, deparaffinized sections were subjected to antigen retrieval and then incubated in 3% H_2_O_2_ for 10 min. Goat serum was used to block non-specific antibody binding. The sections were then incubated with primary antibodies against platelet endothelial cell adhesion molecule-1 (anti-CD31, rabbit monoclonal to CD31; Abcam, Britain) and dentin sialophosphoprotein (anti-DSPP, rabbit polyclonal to DSPP; Bioss, China) at 4°C overnight. Immunolabeling was performed using a horseradish peroxidase-conjugated anti-rabbit secondary antibody (MXB Biotechnologies, China). A DAB substrate kit was used for chromogenic detection and the nuclei were counterstained with hematoxylin. Images were recorded using an Olympus BX53 microscope. Three representative fields from three independent samples in each group were quantified to calculate the positively stained areas or the number of blood vessels.

### 2.12 Statistical analysis

Statistical analysis was performed using an independent Student’s *t*-test or analysis of variance with Prism 9.0 software (GraphPad Software Inc., United States). Values in all experiments were presented as mean ± standard deviation and *p* < 0.05 was considered statistically significant.

## 3 Results

### 3.1 Evaluation of the decellularization procedure

**FIGURE 2:**
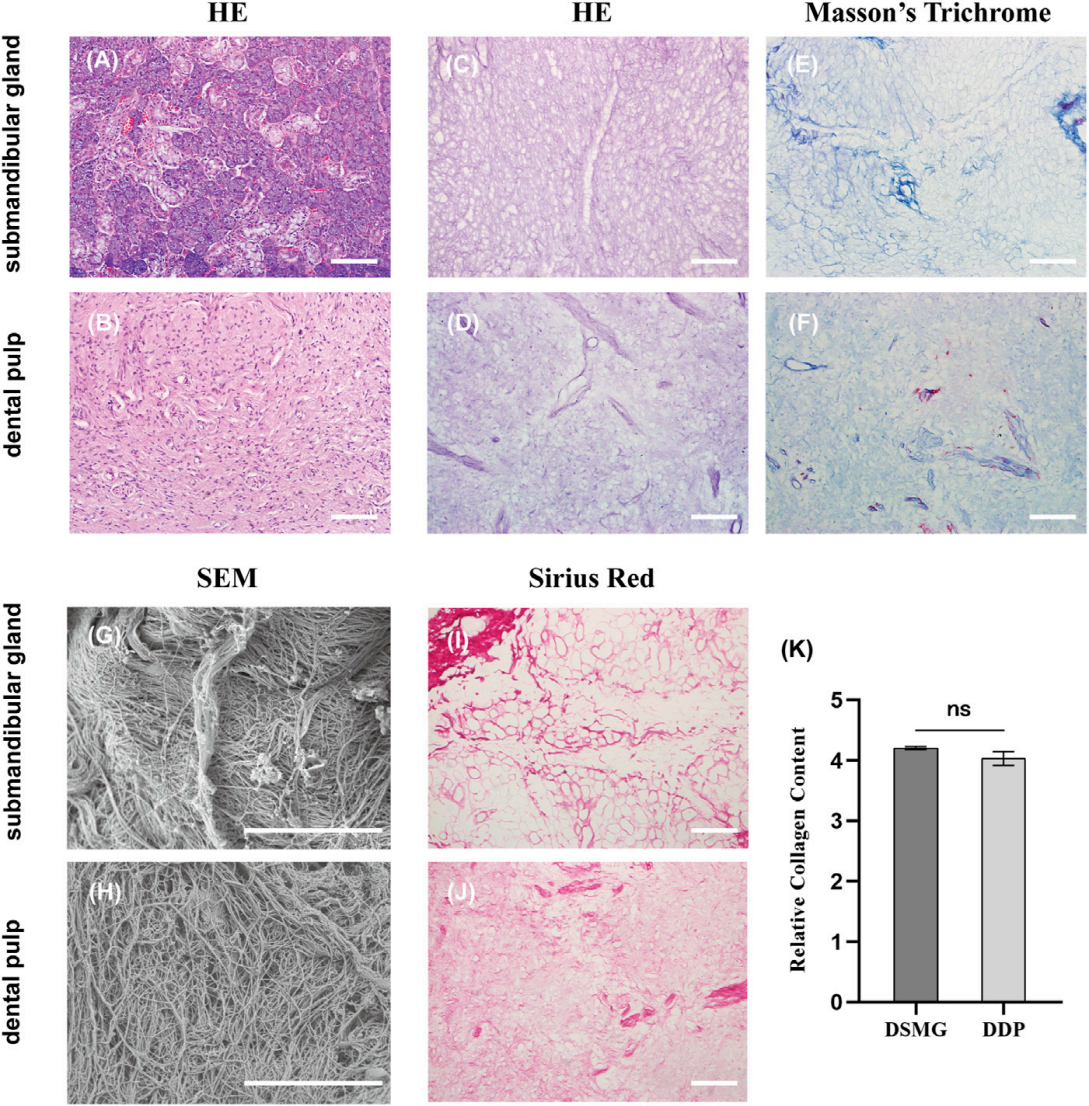
Characterization of ECM in the submandibular gland and dental pulp. **(A,B)** show H&E staining of native submandibular gland and dental pulp; **(C,D)** show H&E staining of DSMG and DDP; **(E,F)** show Masson’s trichrome staining of DSMG and DDP; **(G,H)** show SEM analysis of DSMG and DDP; **(I,J)** show Sirius red staining of DSMG and DDP; **(K)** shows the calculation of relative collagen content, revealing no significant difference in the amount of collagen between DSMG and DDP. ECM, extracellular matrix; H&E, hematoxylin and eosin staining; DSMG, decellularized submandibular gland; DDP, decellularized dental pulp; SEM, scanning electron microscope. Scale bars: 100 μm in **(A–F,I,J)**; 10 μm in **(G,H)**.

Decellularization was deemed successful when no nuclei were visualized in the DSMG and DDP (Figures 2A–D). Furthermore, removal of the cellular contents with retention of native ECM histoarchitecture and vasculature following decellularization was verified using H&E and Masson’s trichrome staining (Figures 2C–F).

### 3.2 Characterization of the ECM structure and components of the submandibular gland and dental pulp

**FIGURE 3:**
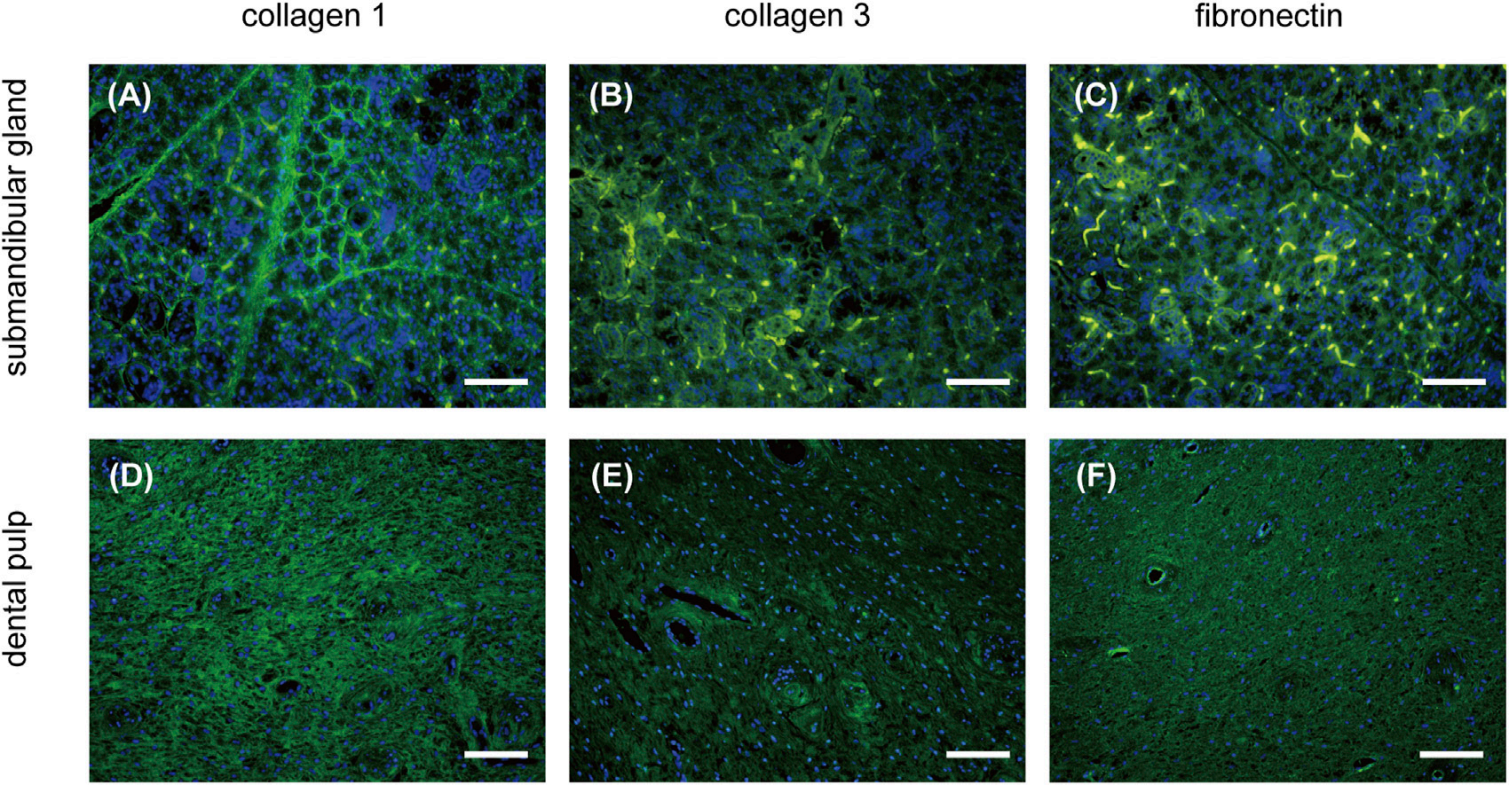
IF characterization of ECM composition of the submandibular gland and dental pulp. The expression of COL1, COL3, and fibronectin (green) in the submandibular gland is presented in **(A–C)**, respectively. The expression of COL1, COL3, and fibronectin (green) in the dental pulp is presented in **(D–F)**, respectively. IF, immunofluorescence; COL1, collagen type I; COL3, collagen type III. Scale bars: 100 μm.

Immunolabeling of COL1, COL3, and fibronectin revealed comparable ECM components in the native submandibular gland and dental pulp (Figure 3). COL1 was observed in a reticulate pattern throughout the matrix in both tissues (Figures 3A, D), whereas COL3 was scattered on the periphery of acini and ducts in the submandibular gland (Figure 3B). Fibronectin was found in the dental pulp matrix with enhanced perivascular expression (Figure 3F).

Sirius red staining confirmed preservation of the collagen network in the decellularized ECM (Figures 2I, J). In addition, semi-quantitative analysis of stained sections revealed no significant difference (*p* = 0.06) in total collagen content between the DSMG and DDP (Figure 2K). The ECM of DSMG presented a loose, fibrous network structure similar to that of DDP, as shown in SEM micrographs (Figures 2G, H).

### 3.3 DSMG could support adhesion and proliferation of DPSCs *in vitro*

**FIGURE 4:**
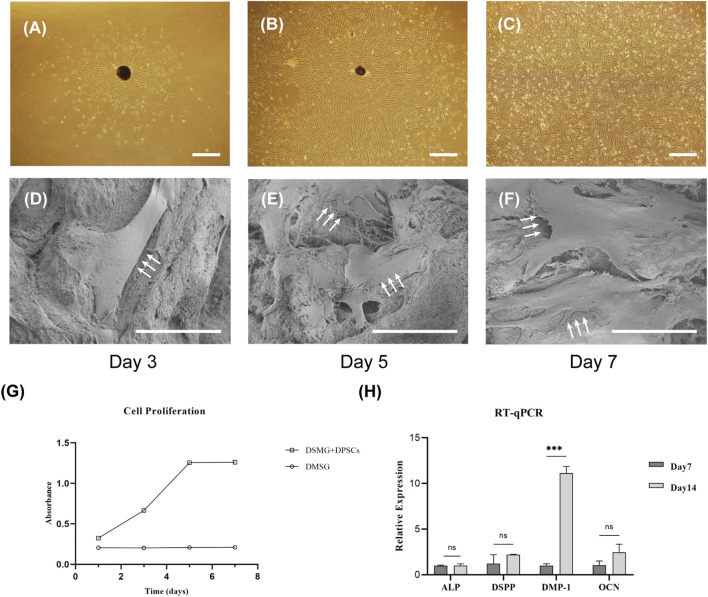
The adhesion, proliferation, and differentiation of DPSCs in scaffolds *in vitro*. **(A–C)** shows the primary culture and subculture of DPSCs. **(D–F)** shows SEM images of DPSCs adhering to scaffolds harvested on days 3, 5, and 7. White arrows point to DPSCs with numerous cytoplasmic processes. **(G)** shows the proliferation of DPSCs on the scaffold over time. **(H)** shows the evaluation of the expression of genes involved in odontoblastic differentiation by RT-PCR. Data are presented as mean ± standard deviation. ***p < 0.001. DPSC, dental pulp stem cell; RT-qPCR, Real-time quantitative polymerase chain reaction. Scale bars: 50 μm in **(A–C)**; 30 μm in **(D–F)**.

DPSCs were successfully obtained and showed a spindle morphology in culture dishes (Figures 4A–C). SEM micrographs demonstrated that DPSCs adhered to the DSMG scaffolds with numerous cytoplasmic processes spreading outward (Figures 4D–F). The DPSCs seeded in the DSMG scaffolds showed continuous viability over 7 days *in vitro* and continued to grow within 5 days of initiation, with the cell number remaining constant thereafter (Figure 4G).

### 3.4 Effect of DSMG on the differentiation potential of DPSCs

Real-time PCR was performed to evaluate the effect of DSMG on the differentiation potential of DPSCs. *DSPP* has been reported to be highly expressed in odontoblasts, although it can also be found at low expression levels in osteoblasts (Qin et al., 2002). *DMP-1* expression was noted in differentiating odontoblasts during early dentinogenesis, but it gradually downregulated in mature odontoblasts. We observed that the expression of *DMP-1* in DPSCs grown in the DSMG scaffold was significantly higher on day 14 than on day 7, but that of *ALP*, *DSPP*, and *OCN* showed no significant increase, which indicated that DSMG alone failed to induce odontogenic differentiation of DPSCs (Figure 4H). One possible reason for the increase in DMP-1 could be due to the three-dimensional structure and ECM cues provided by DSMG to support optimal cell-matrix interactions. However, the possible mechanism remains to be further investigated.

### 3.5 *In vivo* dental pulp regeneration

**FIGURE 5:**
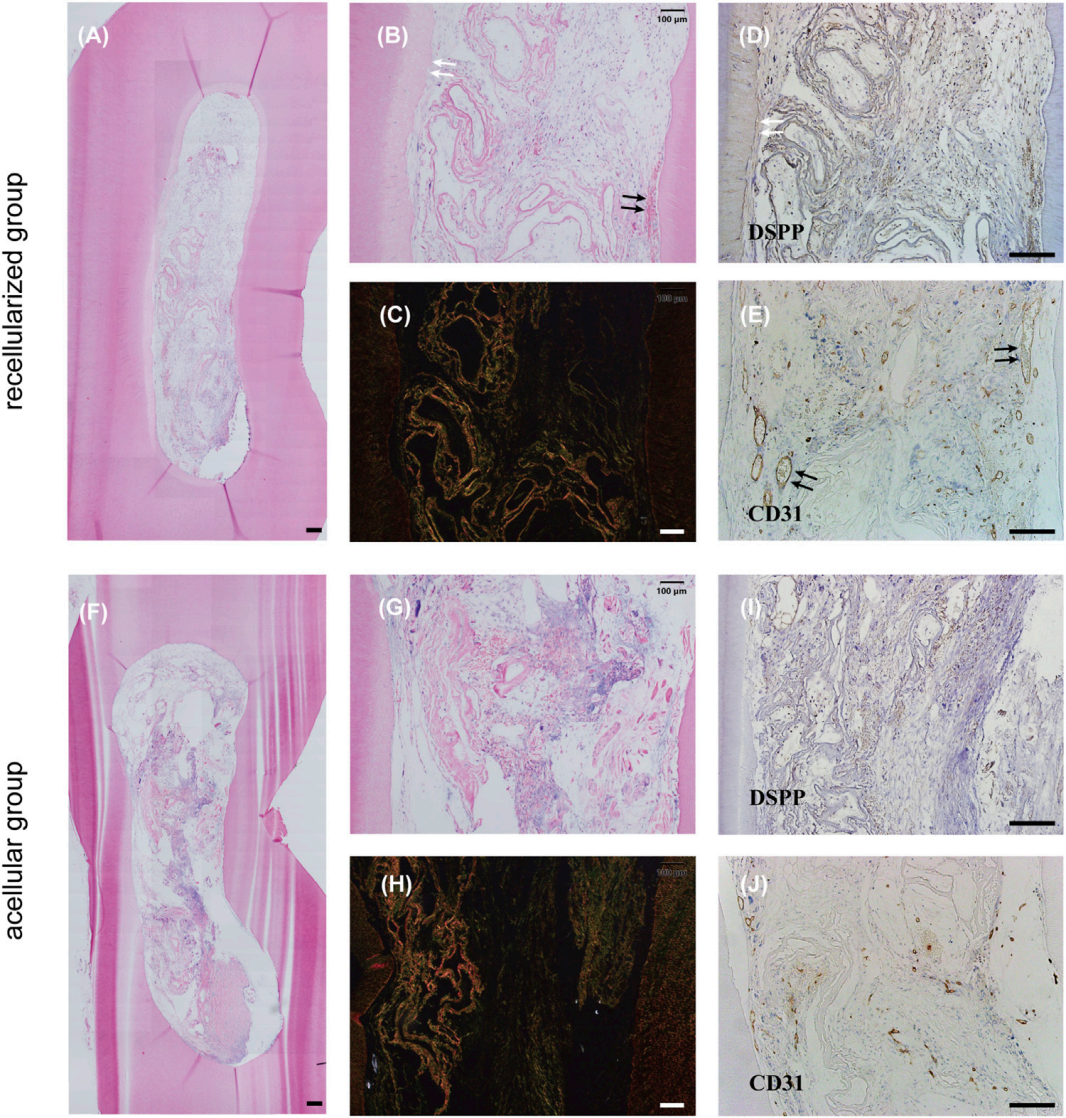
Evaluation of *in vivo* dental pulp regeneration. **(A,B,F,G)** show H&E staining of the newly formed tissue *in vivo*. White arrows in **(B)** point to the newly secreted dentin layer and black arrows in **(B)** point to blood vessels. **(C,H)** show Sirius red staining. **(D**,**E,I,J)** show IHC staining for DSPP **(D,I)** and CD31 **(E,J)**. White arrows in **(D)** label positive reaction around newly secreted dentin and black arrows in **(E)** label positive reaction around blood vessels. **(A–E)** recellularized group; **(F–J)** acellular group. H&E, hematoxylin, and eosin staining; IHC, immunohistochemical; DSPP, dentin sialophosphoprotein; CD31, platelet endothelial cell adhesion molecule-1. Scale bars: 200 μm in **(A,D,E,F,I,J)**; 100 μm in **(B,C,G,H)**.

Evidence of new tissue formation was found in both groups (Figures 5A–C, F–H), whereas only the recellularized scaffolds facilitated formation of abundant functional blood-perfused vessels in the matrix (Figures 5A, B, E). In addition, the DPSC-seeded group showed cellular infiltration throughout the matrix, with a portion of thin, flattened cells lining the lumina of vasculature (Figures 5A, B, E). A newly secreted, distinct layer of dentin was visible at the periphery of the tissue, and it was indicated that flattened cells near the existing dentin wall differentiated into odontoblast-like cells (Figures 5A, B, D). Intense brown staining around blood vessels appeared in newly formed tissue in the experimental group labeled with CD31 (Figure 5E). The recellularized group showed a significantly higher number of perfused and CD31-positive vessels compared with acellular group, confirming the capacity of DSMG to reestablish vascularization and the contribution of DPSCs to promote constructive remodeling in the regenerated tissue (Supplementary Figures S1A, B; Figures 5E, J). Meanwhile, immunohistochemical staining for antibodies against DSPP showed that DSMG successfully provided an essential ECM microenvironment for dental pulp-dentin regeneration (Figure 5D; Supplementary Figure S1C). Negative immunoreactivity for DSPP was observed in the control group (Figure 5I).

## 4 Discussion

In the present study, we developed a novel biological ECM scaffold to be used in dental pulp regeneration, which was obtained through decellularization of the rat submandibular gland. To investigate the regenerative capacity of the DSMG scaffolds, we implanted constructs containing lyophilized DSMG reconstituted with the cell suspension or with culture medium only (as controls) together with treated tooth slices into immunodeficient mice. New tissues were formed within the root canal in both groups, but only the cell-seeded group showed CD31-and DSPP-positive tissue, indicating the formation of a functional vascular structure and the pulp-dentin complex. Our results suggested that it is practicable to use DSMG as an accessible scaffold for cell adhesion, proliferation, and differentiation to achieve functional pulp tissue regeneration.

Great advances have been made in tissue engineering techniques and tissue engineering-based dental pulp regeneration is expected to replace the current endodontic treatment by forming a physiologically functional pulp-dentin complex (Albuquerque et al., 2014; Xie et al., 2021). In regenerative medicine, it is pivotal to create a biocompatible scaffold to provide effective structural support and replicate the natural environment for cell growth and differentiation (Huang et al., 2021). Various natural and synthetic scaffolds with delivery systems and vascular structures have been designed and fabricated to be used in tissue engineering for dental pulp regeneration with efforts to simulate the native dental pulp environment and thus reconstruct the stem cell niche (Huang et al., 2018; Fu et al., 2020; Li et al., 2020; Li et al., 2021; Bakhtiar et al., 2022). Among these solutions, ECM-based biological scaffolds exhibit huge advantages because they are readily available with bioactive molecules and the technology is not sophisticated and less demanding (Badylak et al., 2009). Moreover, decellularization is a viable option to generate the ECM of native tissue and anticipated to provide a routine procedure for eliminating immunogenicity while retaining fundamental matrix components (Zhang et al., 2022). The mechanical properties of decellularized scaffolds changed during the *in vivo* remodeling process, as did the release of inherent bioactive components through scaffold degradation, together with new ECM deposition involved in such biological activities. Our results revealed that the implanted engineered constructs gradually degraded *in vivo*, along with cellular infiltration and new collagen deposition throughout the matrix, and were eventually replaced by newly formed, highly vascularized connective tissue without macrophages, lymphocytes, or other chronic inflammatory cells.

Collagen is a predominant component in native dental pulp, mostly comprising COL1 and COL3 (Linde, 1985). Fibronectin is also found in a reticular pattern throughout the dental pulp matrix with dense concentrations around blood vessels and acts as a mediator of cell adhesion and cell-matrix interactions (McDonald, 1988; Sechler et al., 1998). It is noteworthy that the DSMG retained essential ECM components, such as collagen, fibronectin, and laminin, as demonstrated in our previous study, which are akin to the above-mentioned major components of dental pulp ECM (Gao et al., 2014). Previous studies have successfully obtained xenogeneic DDP scaffolds from human, swine, or bovine dental pulp and thoroughly characterized pulp-derived ECM for dental pulp regeneration (Song et al., 2017; Zhang et al., 2017; Alqahtani et al., 2018; Matoug-Elwerfelli et al., 2018; Bakhtiar et al., 2020; Alghutaimel et al., 2021; Bakhtiar et al., 2021). However, these studies indicated that it is hard to collect abundant DDP scaffolds even using large animal models. As previously mentioned, the basic problem lies in the limited volume and quantity of DDP scaffolds developed for clinical applications. Hence, we introduced DSMG, a decellularized ECM from submandibular glands, as a novel approach. Freeze-drying has so far been suggested to preserve decellularized grafts, allowing for cost-effective storage and easy transport without altering the early hemodynamic performance and biological functions of decellularized scaffolds *in vivo* (Goecke et al., 2018; Zouhair et al., 2019). Therefore, we adopted freeze-drying prior to DSMG recellularization. The results of cell growth and differentiation in the scaffolds indicated that decellularization and lyophilization had no destructive effect on bioactivities of the ECM.

To enhance revascularization is of great importance in regenerative engineering for supporting the survival of implanted cells and, in view of this, strategies to promote revascularization of the engineered constructs determine the success of dental pulp regeneration (Dissanayaka et al., 2014; Katata et al., 2021; Siddiqui et al., 2021). Previous studies have shown that the vascular structure is well preserved in DSMG and that DPSCs have pro-angiogenic properties through secreting angiogenic growth factors and directly differentiating into endothelial cells (Sui et al., 2019). Taken together, these qualities have shown promise in angiogenesis for dental pulp regeneration and have further contributed to the anastomosis with host vasculature following *in vivo* implantation, as shown by our *in vivo* findings that blood-perfused vessels were observed in the newly formed tissue and the immunolabeled CD31-positive cells were found around the vascular lumina in the DPSC-seeded group (Sasaki et al., 2020).

Surprisingly, implantation of treated tooth slices filled with recellularized constructs produced highly organized pulp-like tissue, with newly secreted dentin on the inner side of the existing dentin wall. In contrast to the columnar appearance of odontoblasts during active dentinogenesis (Couve et al., 2013), flattened odontoblast-like cells observed in this study were supposed to be responsible for the formation of new dentin on the surface of the pulp tissue, while the cell bodies containing hematoxylin-stained nuclei were enclosed within the biomineralized dentin matrix as opposed to the natural dental tissue (Linde and Goldberg, 1993). Based on previous studies, we speculated that growth factors liberated from dentin, a reservoir of bioactive molecules, could contribute to the enhanced expression of markers involved in odontogenic differentiation (Li et al., 2011; Sadaghiani et al., 2016). This means that the DSMG scaffold has good compatibility and synergistic reaction with natural dentin. However, the limitation of this study is mainly that the mechanism by which ECM regulates polarization and differentiation of DPSCs remains unclear, and further investigation is required to fully understand the matrix remodeling process and to attempt to modify ECM biomaterials to manipulate cell fate (Casagrande et al., 2010; Chen and Liu, 2016; Chang et al., 2021). Nonetheless, DSMG has greatly attracted our interest due to its good structural, biochemical, and biological properties for supporting cells.

## 5 Conclusion

In conclusion, we introduced DSMG as a novel alternative scaffold to regenerate the complex, highly organized dental pulp and restore its physiological functions. Moreover, the present study sheds light on the promise of ECM-based scaffolds for their biological properties and provides a new method to develop off-the-shelf scaffolds for clinical regenerative therapy. Further research, however, is required to gain more insights into the biology of the pulp-dentin matrix**.**

## Data Availability

The raw data supporting the conclusion of this article will be made available by the authors, without undue reservation.
