# Supplementary material for: Decellularized rat submandibular gland as an alternative scaffold for dental pulp regeneration
Source: Front Bioeng Biotechnol. 2023 Apr 21;11:1148532. doi: 10.3389/fbioe.2023.1148532 (PMC10160494; doi:10.3389/fbioe.2023.1148532)
Supplement: Supplementary file 1 [file DataSheet1.docx]

Supplementary Material

Decellularized Rat Submandibular Gland as an Alternative Scaffold for Dental Pulp Regeneration

**Yuanyuan Shi, Yingxin Wang, Zhenhua Gao****^*^, Zhaochen Shan^*^**

*** Correspondence:** Zhenhua Gao: zhenhua_gao@ccmu.edu.cn; Zhaochen Shan: shanzhch629@163.com

# Supplementary Table

**Supplementary Table 1 |** Primers of genes for RT-qPCR

| **Gene name** | **Forward primer** | **Reverse primer** |
| --- | --- | --- |
| ALP | GGACCATTCCCACGTCTTCAC | CCTTGTAGCCAGGCCCATTG |
| DSPP | CGACATAGGTCACAATGAGGATGTCG | TTGCTTCCAGCTACTTGAGGTC |
| DMP-1 | CGTGGACAAAGAAGATAGCAACTCCACG | TTCCGGCTCTCTATCTCAATGTTT |
| OCN | CACACTCCTCGCCCTATT | GGTCTCTTCACTACCTCGCT |
| GAPDH | TCATGGGTGTGAACCATGAGAA | GGCATGGACTGTGGTCATGAG |

# Supplementary Figure


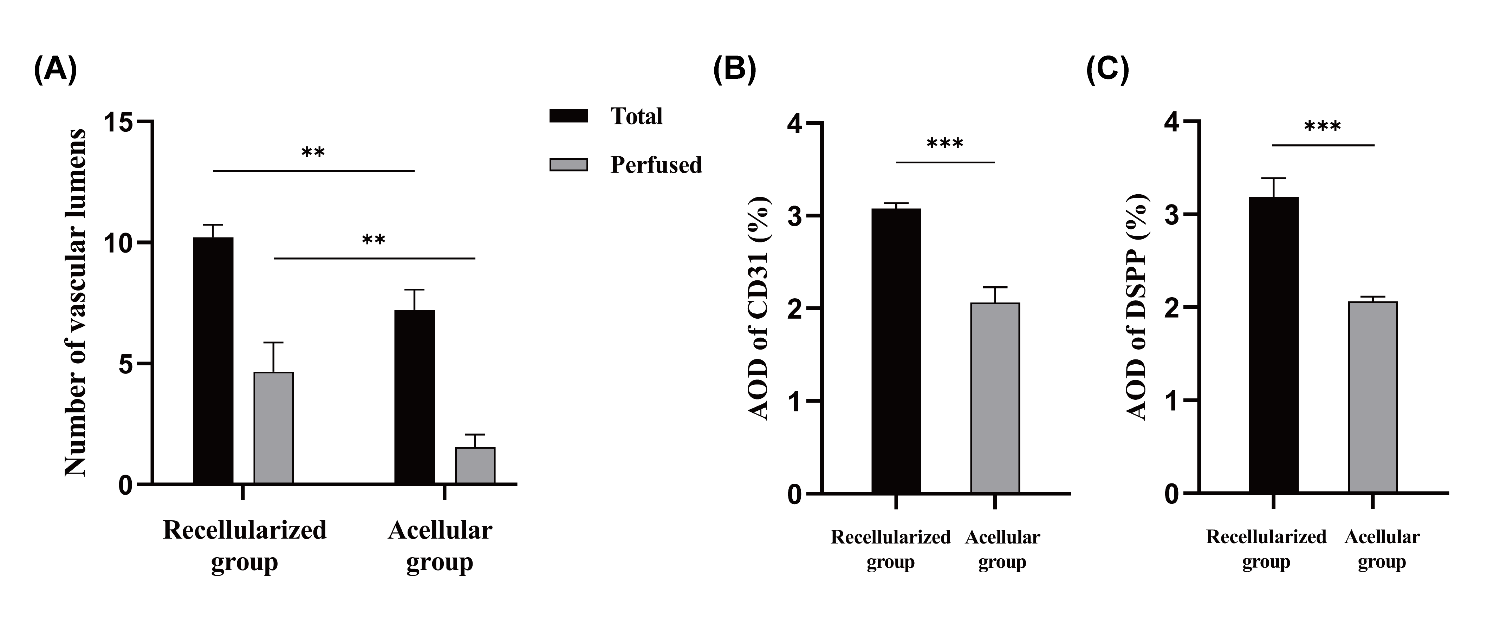


**Supplementary Figure 1 |** Quantified results of regenerated pulp-like tissue in the recellularized and acellular groups. **(A)** shows the number of total vascular lumens and perfused vascular lumens. **(B)** shows CD31-positive staining area. **(C)** shows DSPP-positive staining area. Data are presented as mean ± standard deviation. ***P* < 0.01; ****P* < 0.001. AOD, average optical density.
